# Robust Radiation Sources Localization Based on the Peak Suppressed Particle Filter for Mixed Multi-Modal Environments

**DOI:** 10.3390/s18113784

**Published:** 2018-11-05

**Authors:** Wenrui Gao, Weidong Wang, Hongbiao Zhu, Guofu Huang, Dongmei Wu, Zhijiang Du

**Affiliations:** 1 State Key Laboratory of Robotics and System, Harbin Institute of Technology, Harbin 150001, China; wenruigao@outlook.com (W.G.); ibiaozi@hotmail.com (H.Z.); wdm@hit.edu.cn (D.W.); duzj01@hit.edu.cn (Z.D.); 2 Zhejiang Province Environmental Radiation Monitoring Center, Hangzhou 310000, China; hgf998@126.com

**Keywords:** particle filter, Bayesian estimation, radiation sources localization, nonparametric estimation, multi-modality maintenance

## Abstract

This paper addresses a detection problem where sparse measurements are utilized to estimate the source parameters in a mixed multi-modal radiation field. As the limitation of dimensional scalability and the unimodal characteristic, most existing algorithms fail to detect the multi-point sources gathered in narrow regions, especially with no prior knowledge about intensity and source number. The proposed Peak Suppressed Particle Filter (PSPF) method utilizes a hybrid scheme of multi-layer particle filter, mean-shift clustering technique and peak suppression correction to solve the major challenges faced by current existing algorithms. Firstly, the algorithm realizes sequential estimation of multi-point sources in a cross-mixed radiation field by using particle filtering and suppressing intensity peak value, while existing algorithms could just identify single point or spatially separated point sources. Secondly, the number of radioactive sources could be determined in a non-parametric manner as the fact that invalid particle swarms would disperse automatically. In contrast, existing algorithms either require prior information or rely on expensive statistic estimation and comparison. Additionally, to improve the prediction stability and convergent performance, distance correction module and configuration maintenance machine are developed to sustain the multimodal prediction stability. Finally, simulations and physical experiments are carried out in aspects such as different noise level, non-parametric property, processing time and large-scale estimation, to validate the effectiveness and robustness of the PSPF algorithm.

## 1. Introduction

With the growing number of aging nuclear facilities and uncontrollable nuclear incidents, the ability to efficiently inspect and localize radioactive materials has become an important prerequisite for rapid rescue missions [1,2,3,4]. Under those circumstances, autonomous mobile robots such as Unmanned Ground Vehicles (UGVs) could be deployed to monitor and locate radiation sources in hazardous area. Although the topic of sources localization has received a fair bit of attention in recent decades [5,6,7], the localization for mixed multi-modal radiation filed formed with several gathered radiation sources (e.g., multi-point nuclear leakage) has not been carefully investigated and resolved yet. Our goal is to develop a practicable and nonparametric method to estimate the source number and parameters (i.e., locations and strength), utilizing sparse measurements collected by a low-cost Geiger-Müller detector.

For localizing multiple radiation sources, various formulations of this problem have been studied extensively [8,9,10,11,12,13,14,15,16,17]. Earlier works only deal with separately distributed sources [8,9,10,11], that is, the radiation field could be characterized by several unimodal distributions. Thus source parameters could be estimated on the basis of the individual distributions, while the cumulative effect of multiple sources has been neglected [11]. Beyond that, mixture models and stochastic processes have also been adopted to locate the sources, incorporating the cumulative effect into these statistic models [12,13,14,15,16,17]. As referenced in [15], Morelande et al. approximates the radiation field as Gaussian mixtures, of which the component number is selected through exhaustive cases by the CRB criterion [16]. A similar BIC criterion is utilized in [10], where mixture models with fixed component number are presented to identify the radiations sources, and then MLE method is employed to estimate parameters. For these number selection based algorithms, a large amount of time has been spent on the exhaustive prediction under different number conditions, feasibility can’t be guaranteed when experimental scene is scaled beyond four sources. In addition, Gaussian Processes [12,13] and Poisson Processes [14] are utilized to predict the mixture of radiation sources, unfortunately these stochastic processes only work well when measurements are collected in high density, which is impossible in vehicle-based detection and sampling situations.

As the source localization procedure can also be deemed as radiation field mapping, we can transform the estimation problem into the issue of radioactive intensity mapping [13,18,19]. In literature [13], the radiation map is divided into a finite number of grid cells, and dense sampling is employed to update intensity for each cell. In contrast to the exhaustive approach, Han et al. presents a topological map to describe the radiation field, whereby spiral trajectory and counter lines are depicted in literature [19]. Another interesting kind of localization algorithm is based on particle filter. Chin et al. [20] induced the notion of fusion range to alleviate the mutual impact of multiple sources, and particle filter coupled with mean-shift technique [21] to estimate source parameters. Although this Monte Carlo sampling method could estimate the sources in a sequential and nonparametric manner, it still can’t solve the mutual effects of multiple sources, especially in a scenario where sources are located in close proximity. Furthermore, the particle filter is poor at consistently maintaining multi-modality for target distribution [22], that is, particles would quickly migrate to one of the modes in estimation procedure. Evidently, the inherent unimodal feature hinders the application of particle filter based methods for the mixed multi-modal field.

Motivated by the above discussion, an estimation formulation combined with multi-layer particle structure, particle weight correction and balance maintenance module has been conceived and developed in this article. The PSPF algorithm handles issues of cumulative effect and non-parametric estimation, and has shown excellent computational performance on dimensional scalability. The main contributions of this paper are illustrated in the following points:

- The PSPF algorithm introduces a multi-layer state structure instead of a unified space, i.e., particle swarm of each layer just identifies a single source. This structural improvement, coupled with sequential estimation and weight suppression operation, can effectively tackle the cumulative effect that detected total dose rate could not be utilized directly for the prediction model. Additionally, as the layer extension is roughly linear with processing runtime, the proposed algorithm shows excellent performance on dimensional scalability while existing methods always fall into dimension disaster problem.
- Besides the observation probabilistic model, peak suppression factor and swarm distance factor are also incorporated into the particles weighting process. The peak suppression technique utilizes the sigmoid function to decrease the weights of particles which possess higher radiation strength. The latter factor is adopted to avoid sequential cluster collapse as the swarms get too close in proximity. These factors can efficiently ensure the multimodal balance and speed up the estimation procedure. Through the synthesized weighting model and sequential multi-layer prediction, the challenges in mixed multi-modal radiation field have been overcome.
- Due to the lack of prior knowledge about source number, it is impossible to locate the sources by pre-defining an accurate source number. In existing algorithms, the ambiguity about source number is solved by exhaustive method [15,23], which is not a practicable and real-time solution. Enlightened by the fact that redundant swarms always remain non-cluster or minimal strength status in prediction procedure (when the swarm number is larger than source number), the non-parametric performance about source number could be obtained by the multi-layer prediction structure in our algorithm, as shown in Figures 6 and 8.
- In the parameter estimation phase, a filtering criterion has been conceived and applied after the mean-shift clustering procedure. This clustering method don’t need to explicitly associate the cluster centroids with individual radiation sources, while the filtering criterion could determine if the cluster centroid is available or not. The above mentioned criterion is helpful to simplify the algorithm structure and improve calculative efficiency.

The structure of this paper is organized as follows: the multi-sources localization problem and measurement model are formulated mathematically in Section 2. In Section 3, several necessary state estimation components are reviewed before presenting the algorithm in detail, consisting of particle filter and mean-shift clustering. Then, the major framework and functional modules of the proposed method are expounded and clarified in Section 4. In the following section, simulation and field experiments coupled with quantitative analysis are provided and discussed. Finally, Section 6 concludes this paper and presents some prospects for future work.

## 2. Problem Statement and Detection Modelling

### 2.1. Problem Statement

**Figure 1:**
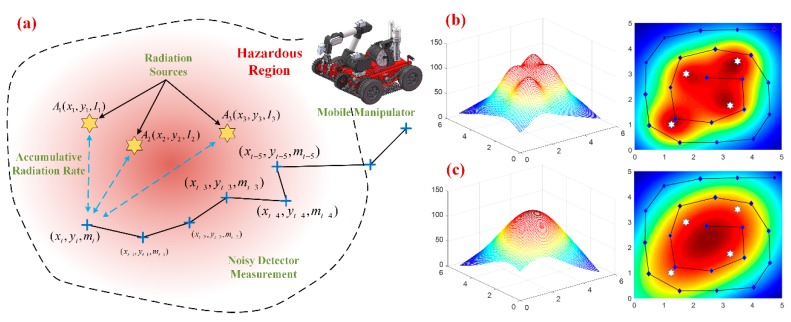
The multi-source radiation scene and problem illustration. (**a**) The scenario illustration of multi-source localization. (**b**) Theoretical radiation strength cloud chart for 4 radioactive sources. (**c**) Radiation strength cloud chart with simply GPs regression.

Our research focuses on how to estimate the number and parameters (locations and strength) of the clustered multiple sources, with the sparse measurements collected by the vehicular GM sensor. As the cumulative effect of radiation intensity, a multi-modality radiation field is formed and estimated without exact prior information about source number and intensity. This setting is applicable and practicable as it can be always encountered in nuclear leakage incidents [1,3], e.g., plant pipeline leakage, transportation accidents, etc. Compared to the existing particle filter based estimation methods [5,20], the main difficulties in the multi-modal radioactive scenario are illustrated as below:

(i) As the GM-type detector could just provide a noisy total dose rate in one location, Poisson based observation model could not be utilized directly in our case. Moreover, the cumulative effect and mixed radiation field invalidate the gradient prediction methods [24] while only an ambiguous hotspot could be perceived. Similar dilemma occurs in mixture models [15,16] as no conjugated distribution exists.
(ii) Considering the limited exploration period and sluggish sensor response, only sparse measurements could be collected by the GM counter. These spatially separate data always fail to construct the multi-modality field by regression methods (e.g., Gaussian Processes), as illustrated in Figure 1c. Furthermore, the peak values of the radiation field don’t coincide with the source locations as the radioactive cumulative effect.
(iii) As the proposed algorithm is formulated on the basis of particle filter, a structure improvement should be performed to realize multi-modality maintenance for the target distribution [22]. Additionally, several measurements should be taken to enable the weighting model to process the collected measurements, which represent the cumulative dose rate in each location.

The multi-source radiation scenario is shown in Figure 1a. It is assumed that the two-dimensional hotspot is formed by several radiation sources in close proximity, while a mobile vehicle armed with GM counter is utilized to detect and estimate radiation sources in the surveillance area. Similar to the settings in [20], let the three-value vector $A_{j}=\{A_{j}^{x},A_{j}^{y},A_{j}^{str}\}$ denotes the location and strength information of the *j*-st source, for 1 ≤ *j* ≤ *K*. The locations $A_{j}^{pos}$ can be measured in meter and radioactive dose rate $A_{j}^{str}$ in Gy/h respectively. As gamma-ray complies with the inverse square propagation [25], the radiation intensity for single source component can be expressed as:

(1) $$I(S,A_{j})=\frac{A_{j}^{str}}{h^{2}+{\left|S-A_{j}^{pos}\right|}^{2}}exp(\sum_{b\in (B\cap \bar{SA_{j}})}-\mu_{b}l_{b})\;$$

where *S* indicates the position of measuring point, and *h* is the height difference between measurement plane and source plane. *B* denotes a set of obstacles, and *l_b_* and *μ_b_* denote the thickness and attenuation coefficient of the obstacles respectively. It could be noticed that no obstacle exists in our setting, as the issue assumption about radioactive cumulating effect.

Considering the limited exploration period and sluggish sensor response, it could be seen that densely sampling radiation field is impracticable in this scenario, instead only spatially sparse measurements can be obtained in the inspection process, as shown in Figure 1b. Different from actual multi-modal distribution in Figure 1b, the regressive distribution with these sparse measurements fails to restore the multi-modality feature of radiation field, demonstrating simple regression [12,13] or direct mapping methods [19,26] are inapplicable in our detection case. All the difficulties motivate us to improve the traditional particle filter, whereby the unified state space has been modified into a multi-layer particle structure, and each layer just identify one radioactive source or not. Additionally, the peak suppression module is incorporated into the particle weighting procedure, which can obtain excellent performance of multi-modality maintenance in the state space. The details of the PSPF algorithm can be seen in Section 4.

### 2.2. Detection Modelling

Instead of the sensor network applications [5,15,16,20], the radiation sensor is mounted on the mobile vehicle, which inspects the hazardous zone through sampling trace points. As the GM-type sensor measures radiation intensity by counting the ionization flux, i.e., counts per second (CPS), the multi-source radiation field can be measured by the cumulative effect of the multiple sources, as expressed in the following:

(2) $$I_{i}=E_{i}\cdot \sum_{j=1}^{K}I(S_{i},A_{j})+B_{i}\;$$

where *I_i_* denotes the total dose rate at the location *S_i_*, and *A_j_* denotes the *j*-st radiation source. *E_i_* represents the conversion constant utilized to convert the unit from CPS to Gy/h in our application. *B_i_* indicates the background radiation which is naturally present due to radio-isotopes.

According to the measuring principle described above, Poisson distribution could be employed to weight the estimated particles if a single source exists. In fact, Poisson distribution is always utilized to handle the counting event whereby expected count is *λ* and actual count is *k*, as illustrated as Equation (3):

(3) $$P(X=k|\lambda )=\frac{\lambda^{k}e^{-\lambda }}{k!}\;$$

Evidently, above Poisson-based weighting model could not be incorporated into the algorithm scheme directly, for the reason that only total radiation intensity is gauged by the GM sensor. The key idea of our solution is to split measurements into individual hypothesis for each particle swarm, which is expounded in detail in Section 4.1 and Section 4.2.

## 3. Preliminary Estimation Methods

### 3.1. Particle Filter

**Figure 2:**
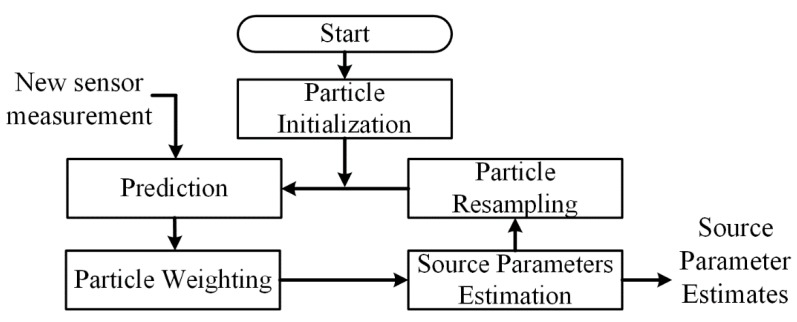
Flowchart of the traditional particle filter in sources localization application.

With the simplicity, universality and extensive applications in localization field, particle filter has become one of the most famous estimation methods in Bayesian estimation. This method discretely depicts target distribution by a set of weighted state particles, and iteratively motivates particles to move towards the actual distribution [27]. The flowchart of particle filter is illustrated in Figure 2. The iterative estimation in particle filter can be divided into two phases: prediction and updating. The updating phase approximates the posterior distribution by inputting the current observation, while prediction step estimates the prior distribution through state transition factor without current observation factor. According to the Bayes formula and Markov hypothesis [28], this sequential Monte Carlo model can be represented as follows:

(4) $$\begin{array}{ll} p(x_{0:t}|y_{1:t}) & \underset{\to }{updating}\int \delta (x_{0:t})\frac{p(x_{0:t}|y_{1:t})}{q(x_{0:t}|y_{1:t})}q(x_{0:t}|y_{1:t})dx_{0:t}\\ & =\int \delta (x_{0:t})\frac{p(y_{t}|x_{0:t},y_{1:t-1})p(x_{t}|x_{0:t-1},y_{1:t-1})p(x_{0:t-1}|y_{1:t-1})}{p(y_{t}|y_{1:t-1})q(x_{t}|x_{0:t-1},y_{1:t})q(x_{0:t-1}|y_{1:t-1})}q(x_{t}|x_{0:t-1},y_{1:t})q(x_{0:t-1}|y_{1:t-1})dx_{0:t}\\ & \underset{\to }{prediction}\eta \cdot p(x_{0:t-1}|y_{1:t-1})\cdot \int_{x_{t}}\delta (x_{t})\frac{p(y_{t}|x_{t})p(x_{t}|x_{t-1})}{q(x_{t}|x_{t-1},y_{t})}q(x_{t}|x_{t-1},y_{t})dx_{t} \end{array}$$

where *p*(*x*_0*:t*_|*y*_1*:t*_) and *p*(*x*_0*:t−*1_|*y*_1*:t−*1_) are the posterior probability for the whole time series until time *t* and *t* − 1. *q*(*x_t_*|*y_t_*, *x_t−_*_1_) indicates the proposal distribution at time t incorporating the particle *x_t−_*_1_, and *δ*(*x_t_*) is dirac function denoting the discrete sampling process for target distribution.

Considering the Bayes formula and Markov hypothesis, the approximation for current state particle *x_t_* can be induced as:

(5) $$\begin{array}{ll} p(x_{t}|y_{t},x_{t-1}) & =\frac{p(x_{0:t}|y_{1:t})}{p(x_{0:t-1}|y_{1:t-1})}\\ & =\eta \cdot \int_{x_{t}}\delta (x_{t})\frac{p(y_{t}|x_{t})p(x_{t}|x_{t-1})}{q(x_{t}|x_{t-1},y_{t})}q(x_{t}|x_{t-1},y_{t})dx_{t}\\ & \approx \sum_{i=1}^{N}\tilde{w}(x_{t}|x_{t-1}^{\left(i\right)})\cdot \delta (x_{t}^{\left(i\right)}) \end{array}$$

where proposal distribution *q*(*x_t_*|*y_t_*, *x_t−_*_1_) is set as transition model *p*(*x_t_*|*x_t−_*_1_), thus particle weight model can be simplified to be $w_{t}(x_{t}^{\left(i\right)})\propto p(y_{t}|x_{t}^{\left(i\right)})\cdot w_{t-1}(x_{t-1}^{\left(i\right)})$. As estimated radiation field is assumed to be quasi-static, the transition model *p*(*x_t_*|*x_t−_*_1_) obeys Gaussian distribution, and the observation model can be represented by Poisson distribution, as shown in Equation (3).

### 3.2. Mean-Shift Clustering

Mean-Shift procedure is a non-parametric clustering approach on the basis of kernel density estimation [21]. Given the current state of particles, the mean-shift method can rapidly estimate the density distribution in state space, and motivates the sampling points towards local maximum, realizing the cluster labelling in the end. Benefiting from the versatile and rapid characteristic, the mean-shift method has been widely employed in multi-modality localization problem [21,29,30], and also provides robust and reliable clustering results for the calculating model. The process of mean-shift clustering is actually an iterative optimization procedure, whereby the state shift *M*(*x*) could be expressed as:

(6) $$\left\{ \begin{array}{l} M(x)=\frac{\sum_{P}w(p_{i})\cdot \phi_{H}(p_{i}-x)\cdot (p_{i}-x)}{\sum_{P}w(p_{i})\cdot \phi_{H}(p_{i}-x)}\\ \phi_{H}(x)={\left(2\pi \right)}^{-3/2}|H{|}^{-1/2}\exp(-\frac{1}{2}x^{T}H^{-1}x) \end{array}\right.\;$$

where $\phi_{H}(x)$ is the Gaussian kernel function used for density estimation, *H* is a positive definite matrix denoting the bandwidth in each dimension, and *M*(*x_i_*) indicates the shift vector pointed to state *x_i_*.

In our application of multi-source localization, several initial states should be randomly sampled in the space, and each sample needs to find the local maximum individually. Thus the global maximum and cluster labelling could be realized through these samples. In contrast to general clustering methods (i.e., GMM, k-means), mean-shift technique is a non-parametric estimation, which is helpful in reducing the burden on parameters tuning and dimensional simplicity. In addition, this method shows a good performance about noisy robustness, which would be discussed in Section 5.

## 4. Proposed Algorithm Design

**Figure 3:**
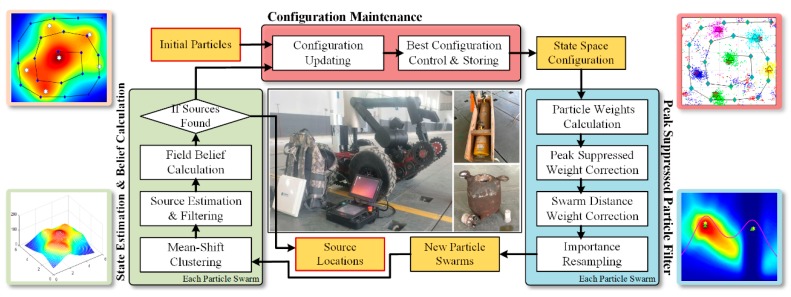
Flow diagram of the proposed PSPF algorithm.

This section mainly describes our proposed algorithm for localizing multiple radiation sources in a cross-mixed environment (i.e., a radiation field of which the strength is affected by several sources), utilizing the dose measurements in different places by vehicular radiation detector. Given a newly acquired or selective measures, the proposed algorithm can refine the location and strength messages for each radiation source. The flowchart of the PSPF algorithm is illustrated as Figure 3.

The algorithm starts with the *particle initialization* for the multi-layer particle swarms, and each particle swarm is utilized to estimate the parameters of a single radiation source. For a new sensor measurement, the above multi-layer particle swarms can be estimated sequentially, that is, the later particle swarm should be predicted on the basis of the estimation results of the previous layers. Furthermore, the particle aggregation process for particle swarm can be summarized into two phases, i.e., *weighting* and *resampling*. The *weighting* step is to approximate the weight for each state particle in current swarm. Compared with the traditional particle filter, this step also takes *peak suppression* factor and *swarm distance* factor into account, to ensure the multi-modality maintenance and estimated stability. Then these weighted particles go through the resampling step to be relocated into higher probabilistic states. After the resampling process, *mean-shift clustering* technique and specific filtering criteria are applied to determine whether a valid centroid exists in current particle swarm. Above estimation procedure (except for particle initialization) should be repeated for all the pre-defined particle swarms, and finally the average confidence score for all existing measurements are approximated to determine whether to continue the estimation loop.

Besides the main estimation procedure, the configuration maintenance has also been conceived and integrated into the proposed scheme. The module works after the confidence calculation step in each iteration. It records the multi-layer configuration with the highest confidence score, which could be restored if the multi-modal balance is broken. In this perspective, the maintenance module is actually a protection mechanism in the prediction procedure.

As expounded above, the proposed algorithm addresses the following problems: (i) the multi-source localization problem in mixed radiation field; (ii) non-parametric estimation about the number of sources; (iii) rapid and steady prediction on individual source parameters implicitly.

### 4.1. Particle Initialization

At initial time, particles in each layer should be initialized as follows. Let $P_{s}=\{p_{r,s}^{\left(t_{i}\right)}|r=1,\cdots ,M_{r}\}$ be the *s*-st particle swarm in the configuration space 𝒫, which is consisted of multiple particle swarms 𝒫*_s_*, for *s* = 1, …, *M_s_*. Similar to previous literatures [5,20], each particle state $p_{r,s}^{\left(t_{i}\right)}$ is a three-value vector denoting source location and strength. The superscript *t_i_* indicates the time step number, and it would be dropped when no ambiguity exists in the prediction procedure. We could initialize each particle swarm 𝒫*_s_* with uniformly random particles, as the reason that no prior knowledge about sources parameters is available. Correspondingly, the particles would be initialized according to specific distribution if prior configuration exists. This procedure is named as configuration maintenance in our algorithm, which has been described in details in Section 4.5.

The number of particle swarms *M_s_* reflects the expected source number in the surveillance area. When the pre-defined swarm number is larger than the actual source number, it could be observed that redundant swarms would always remain non-cluster or minimal strength status in the estimation process. Thus the multiple sources could be estimated in a non-parametric manner. For each particle swarm 𝒫*_s_*, the number of particles given by *M_r_* = |𝒫*_s_*| affects the state diversity of estimated target. That is, a larger particle number will result into a more accurate estimate, otherwise calculation accuracy is reduced. Furthermore, each particle state *p_r,s_*∈*_s_* should be associated with a weight *w*(*p_r,s_*) to measure the probability that an actual radiation source exists in the location *p_r,s_*. However, as the collected measurements only represent total dose rate, measurements should be split into individual hypothesis with support of multi-layer state structure. That is, the estimation for current particle swarm should consider the previous candidated sources located by previous swarms in the weighting step, as shown in Equation (8). The multi-layer particle structure, coupled with the sequential iterative prediction, successfully integrates the multi-source cumulative measurements into the particle filter framework and the Poisson based weighting model.

### 4.2. Synthesized Particle Weighting

After initializing the particles, the corresponding particle weights should be calculated to evaluate the likelihood that an actual radiation source exists in the state. Although there’s no prior information about source number and distribution, the mixed multi-modal field can still be estimated by the aid of multi-layer state structure and sensor measurements. Besides the Poisson observation model (as stated in Section 2.2), peak suppression factor and swarm distance factor are also integrated into the weighting process to realize multi-source localization. The synthesized weighting model can be expressed as follows:

(7) $$w_{syn}^{r,s}=w_{obs}^{r,s}(I_{rand(n)},I_{s,n}^{\prime })\cdot w_{ps}^{r,s}(p_{r,s},\theta_{ps})\cdot w_{dist}^{r,s}(C_{s},p_{r,s})\;$$

In the above equation, synthesized weighting model consists of three components: observation weighting model *w_obs_*, peak suppressed correction *w_ps_* and swarm distance correction *w_dist_*. These three components will be described detailedly in this part.

#### 4.2.1. Detector Weighting Model

Assume that *p_r,s_* is the *r*-*st* particle state in the *s*-*st* particle swarm, and ${\left\{C_{s}\right\}}_{s=1}^{M_{s}}$ denotes the set of candidated centroids for each layer in latest iteration. Then, the observation likelihood for the particle *p_r,s_* given actual measurement *m*(*S_i_*) can be expressed as below:

(8) $$w_{obs}^{r,s}(m(S_{i}),p_{r,s},C_{-s})=\frac{p(m(S_{i})|I^{\prime }(p_{r,s},C_{-s}))}{p(\left\lfloor I^{\prime }(p_{r,s},C_{-s})\right\rfloor |I^{\prime }(p_{r,s},C_{-s}))}\;$$

where *C_−s_* denotes the set of candidated sources exclusive of the current layer centroid. The function *p*(·) denotes the Poisson observation model described in Section 2, whereby the measurement *m*(*S_i_*) is regarded as actual count and expected dose rate is the desired count. Additionally, the normalized constant *p*(⌊*I′*⌋|*I′*) is added to the formulation such that subsequential correction can be conducted in the same scale, and ⌊·⌋ depicts the floor operator.

Benefited from the multi-layer structure, the proposed method embeds the candidated centroids into the Poisson based detection model, and this improvement provides theoretical foundation for the mixed multi-source localization problem, which is infeasible and impracticable in existing research [16,20,31]. Furthermore, this weighting model is adopted alone in the confidence calculation step (as described in Section 4.5), while peak suppressed factor and swarm distance factor are just employed in the weighting process.

#### 4.2.2. Swarm Distance Correction Model

**Figure 4:**
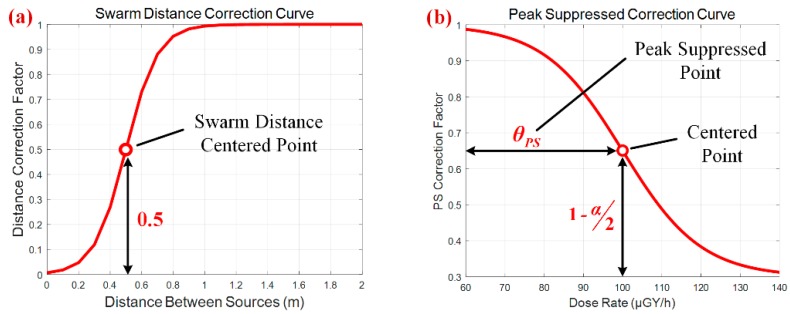
The particle weight correction curves. (**a**) The swarm distance correction curve. (**b**) The peak suppressed correction curve.

In the sequential multi-layer sampling procedure, the collapse of one particle swarm may result in a sequential collapse of the whole estimation structure. This phenomenon always occurs when two particle swarms get too close in proximity, as the existence of one particle centroid can significantly reduce the particle weights in another swarm. To avoid the sequential collapse, sigmoid function is adopted to sharply reduce the particle weights when one particle swarm is close to another swarm centroid. When these particle centroids get separated from each other, the distance correction factor stays near 1.0; otherwise the factor decreases sharply as the distance is smaller than the offset value *θ_dist_*, as illustrated in Figure 4a. The distance correction model is given in the following equation:

(9) $$w_{dist}^{r,s}(p_{r,s},C_{-s})=\frac{1}{1+exp[\frac{\theta_{dist}-dist(p_{r,s},C_{-s})}{b_{dist}}]}\;$$

where *θ_dist_* indicates the offset value of the sigmoid function, *b_dist_* is the scale parameter, and *dist*(·) denotes the minimal distance between current particle and centroids of other swarms.

The introduction of distance correction factor is conducive for maintaining the multi-modality balance of configuration space, and improves the efficiency and stability of the proposed algorithm. In the simulations without correction factor, it could be frequently observed that one particle swarm is replace by another one, then an overall collapse occurs in the estimation procedure. That is, the absence of distance correction factor will definitely result into estimation failure due to frequent breakout of the balance. In this perspective, although this correction factor is a small trick, it seems to be essential for the multi-layer state structure and our proposed algorithm.

#### 4.2.3. Peak Suppressed Correction Model

As discussed above, the independent application of multi-layer structure can’t achieve the expected multi-modality effect, but companied with the peak suppressed correction module to do so. The peak suppression module utilizes the sigmoid function to decrease the weights of particles which possess higher radiation strength, preventing the existence of unimodal estimate with extremely high strength in the state space. The peak suppressed correction model can be expressed as below:

(10) $$w_{ps}^{s,r}(p_{r,s},\theta_{ps})=(1-\alpha )+\alpha \cdot \frac{1}{1+exp[(p_{r,s}^{str}-\theta_{ps})/b_{ps}]}\;$$

where *α* indicates the vertical tuning parameter for the median point, of which the correction is defined as 1−*α*/2. *θ_ps_* is the offset value of sigmoid function, which is also named as suppressed point in our algorithm, and *b_ps_* denotes the scale parameter. By appropriately tuning suppressed point *θ_ps_* and scale parameter *b_ps_*, the PSPF algorithm can obtain an excellent and robust performance about multi-modality maintenance. According to test experience, the suppressed point *θ_ps_* can be set as the 90% of the maximum measurement, and the scale parameter *b_ps_* is generally 10%~20% of the space range. The peak suppressed correction curve is illustrated in Figure 4b, where the suppressed point is set to be 0.7.

**Figure 5:**
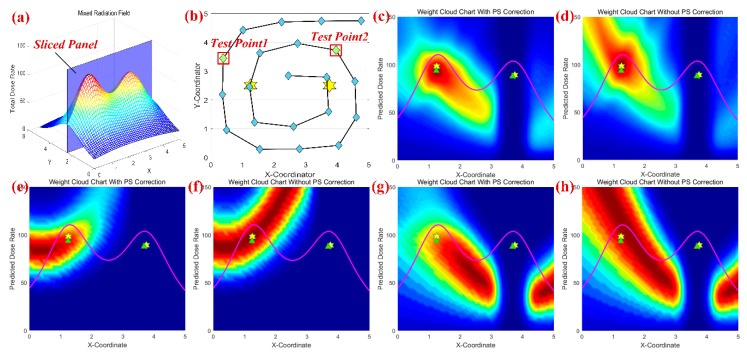
The functional specifications of peak suppressed module. (**a**,**b**) indicates the positions of measurements and sources, and the radiation distribution. (**c**,**d**) shows the overall weight distributions in sliced panel for corrected and non-corrected case. (**e**–**h**) shows the weight distributions for one measurement (point1 & point2) in corrected and non-corrected cases.

The peak suppressed module is conceived to solve the multi-source estimation problem by correcting particle weights with higher radiation strength. To clarify the mechanism of peak suppressed module, a simulation with two radioactive sources has been performed by the PSPF algorithm. The simulation scene and weight cloud chart are represented in Figure 5. For convenience of illustration, two sources are located in a sliced vertical plane, i.e., (1.25, 2.5, 98) and (3.75, 2.5, 90), as shown in Figure 5a. After the estimation process, the particle swarms finally locate at (1.24, 2.53, 93.44) and (3.68, 2.44, 88.68), with the confidence score 97.62%. To show the effect of peak suppressed module, the right estimated centroid is fixed while the left estimate is assumed to be any location in the plane. Then the weight cloud chart for the left centroid can be calculated and obtained, as shown in Figure 5c–h. Figure 5c,d illustrate the average weight distributions for overall measurements in the corrected and non-corrected case. It can be seen that traditional models obviously prefer the locations with higher strength, while the peak suppressed module effectively restrains this strength preference that may destroy the multi-modality balance.

Furthermore, above strength preference could be observed more clearly in the sequential prediction process, whereby only one measurement is approximated in one iteration, the selected test measurements are indicated in Figure 5b. Figure 5f,h have shown the weight distributions in the non-corrected case, it can be clearly seen that there exists several locations with higher weight and strength. As stated earlier, the estimation imbalance can be traced back to inherent unimodal performance of traditional particle filter. This high intensity area will motivate the estimated particles to stay or drive towards these locations, and finally lead to a unimodal distribution. On the other side, the weight cloud charts with the peak suppressed correction can be seen in Figure 5e,g. The obvious difference with the non-corrected case is that weights in high intensity area have been suppressed and reduced, ensuring the availability of sequential multi-layer prediction in the PSPF algorithm.

### 4.3. Importance Resampling

Importance sampling process is a common practice in Sequential Monte Carlo methods to replace particle with low weights to ones with higher weights [27]. The resampling step serves two-fold purpose: (i) as the iteration progresses, resampling step moves the particles towards the states where radioactive sources are most likely to exist, providing the operational basis for the sequential estimation; (ii) after the resampling step, particles should be assigned with the same weights to prevent particle degeneration and maintain state diversity in practice.

Resampling procedure is accomplished by discretely sampling particles from each particle swarm 𝒫*_s,n_* with normalized weights ${\tilde{w}}_{syn}(p_{r,s})$. As the infrastructure of multiple particle swarms in the PSPF algorithm, the resampling procedure with subsequential parameter estimation should be carried out immediately after particle weighting for each particle swarm. This arrangement aims to maintain the multi-modality state in configuration space, as well as to ensure the effectiveness of multi-layer sequential estimation. Additionally, several specific hints should be implemented for resampling step in practice: (i) A tiny zero-mean Gaussian noise should be added to the resampled particles, to prevent particle degeneration in the state space. (ii) A certain proportion (i.e., 5~10%) of particles should be generated with random distribution, to prevent the low space coverage in later estimation phase when all particles are around the candidated sources.

### 4.4. Sources Parameters Estimation

Following resampling step, a state estimation module is employed to predict the candidate source parameters through appropriate clustering technique and filter criterion. In contrast to traditional particle filter, the proposed algorithm generates source parameter estimates from each particle swarm by clustering technique firstly, then the biggest cluster centroid is assigned as the state estimate of current particle swarm. It should be noted that the filtering criterion is utilized to determine whether the centroid is a valid estimate, i.e., the particle number of biggest cluster should be larger than percentage threshold *THR_pr_*, and the radiation intensity of centroid larger than intensity threshold *THR_str_*.

The implementation details of mean-shift technique have been discussed in Section 3.2. This versatile and rapid clustering method makes possible the efficient global optimization and sequential multi-layer estimation. Moreover, the characteristic that cluster centroid could implicitly estimate any radiation source ensures the simplicity of proposed framework, and Gaussian based KDE method also supplies the robust performance against measurement noise.

### 4.5. Configuration Maintenance

The last step of the algorithm, configuration maintenance module, records the particle states and estimated parameters with the highest confidence condition, to the effect that all the particle states could be restored to optimal configuration when multi-sources balance has been broken. Considering the imbalance nature of the multi-source scene, a large estimated deviation may cause a chain-collapse of the multiple particle swarms. During the prediction process, the estimation configuration with optimum field confidence would be persisted all along and updated until a better configuration emerges. Furthermore, a restore operation should be performed when current field confidence has been below the optimum value five times in a row, as to maintain the unstable balance. As stated above, the configuration maintenance step is actually a protection mechanism, which is utilized to avoid previous estimation procedure going to waste due to cluster collapse.

In the maintenance procedure, the calculation of radiation field confidence is essential and accomplished by Poisson distribution model with all the sensor measurements, as expressed in the following:

(11) $$F=\frac{1}{N}\sum_{i=1}^{N}w_{obs}(m(S_{i}),{\left\{C_{s}\right\}}_{s=1}^{M_{s}})=\frac{1}{N}\sum_{i=1}^{N}\frac{p(m(S_{i})|I^{\prime }(S_{i},{\left\{C_{s}\right\}}_{s=1}^{M_{s}}))}{p(\left\lfloor I^{\prime }(S_{i},{\left\{C_{s}\right\}}_{s=1}^{M_{s}})\right\rfloor |I^{\prime }(S_{i},{\left\{C_{s}\right\}}_{s=1}^{M_{s}}))}\;$$

where ${\left\{C_{s}\right\}}_{s=1}^{M_{s}}$ denotes the estimated centroids for each particle swarm, and the definitions of *w_obs_*(·), *p*(·) and *I’*(·) are similar to Equation (8). The confidence score is the average observation weight for all the collected measurements, and this indicator could be utilized to measure the credibility of current state configuration. Then the configuration updating and restoring operations can be determined on the basis of indicator.

### 4.6. The Complete Algorithm

As expounded above, the PSPF algorithm goes through several steps i.e., particle initialization, synthesized particle weighting, importance resampling, sources estimation and configuration maintenance, and realizes multiple sources estimation in the strongly coupled radiation field. The pseudo code of the PSPF algorithm is shown in Algorithm 1.

**Algorithm 1**. Specific Flowchart of the Cross-mixed Multiple Radiation Sources Localization Algorithm based on Peak Suppressed Particle Filter (PSPF)**Inputs:** A sequential measurements of the radiation sensor $I={\left\{I_{n}\right\}}_{n=1}^{N}$, consisting of the total dose rate and their corresponding positions.**Outputs:** State set of the predicted radiation sources $A={\left\{A_{k}\right\}}_{k=1}^{K}$, each state is a three-vector value consisting of position and intensity messages.
**Notes:**
${\left\{C_{s,n}\right\}}_{s=1}^{M_{s}}$ denotes the clustered centroids for each swarm. ${\left\{B_{s,n}\right\}}_{s=1}^{M_{s}}$ denotes the temporal state set for each swarm, as shown in *L*14~*L*18. ${\left\{p_{s,n}^{r}\right\}}_{r=1}^{M_{r}}$ indicates the set of particle states for each swarm. *θ_ps_* and *θ_dist_* are the sigmoid offset value in peak suppression model and distance weighted model respectively.1.**for***s* = 1, …, *M_s_*
**do**▷ **Model Initialization**2.    **for**
*r* = 1, …, *M_r_*
**do**▷ randomly sample the three-value vectors from state-space and assign them as predicted particles.3.        $p_{r,s}^{pos}$~*rand*(*pos_min, pos_max*); 4.        $p_{r,s}^{str}$~*rand*(*str_min, str_max*);5.    **end for**6.
**end for**
7.**for***t* = 1, …, *T*
**do**
**▷ Sources Localization Starting**
8.    Initialize the measurement set I*_active_* = I

9.    **for**
*n* = 1, …, *N*
**do**▷ iteration for each measurement10.        **Select** one measurement *I_rand_*_(*n*)_ from active set I*_active_*
11.        **for**
*s* = 1, …, *M_s_*
**do**▷ iteration for each particle swarm12.            **for**
*r* = 1, …, *M_r_*
**do**
**▷ Peak Suppressed Particle Filter**
13.                **for**
*s’* = 1, …, *M_s_*
**do**▷ construct temporal state set for each particle ${\left\{B_{s,n}\right\}}_{r=1}^{M_{r}}$, consisting of current particle state and centroids for other swarm.14.                  **if**
*s*’ = *s*
**then**15.                        $B_{s^{\prime },n}=p_{s,n-1}^{r}$16.                    **else**
*s*’ ≠ *s*
**then**17.                        $B_{s^{\prime },n}=C_{s^{\prime },n-1}$18.                    **end if**19.                **end for**
20.                **Compute** the predicted dose rate for current position:
**▷ Synthesized Importance Weighting**
21.                    
$I_{s,n}^{\prime }=\mathrm{intensity}\_\mathrm{prediction}(B_{n},I_{rand(n)}^{pos})$
▷ according to expression (2)22.                **Compute** the synthesized weight for each particle:▷ according to expression (9), (10), (11) and (12)23.                    
$w_{syn}^{s,r}=w_{obs}^{s,r}(I_{s,n}^{\prime },I_{rand(n)})\cdot w_{ps}^{s,r}(p_{s,n-1}^{r},\theta_{ps})\cdot w_{dist}^{s,r}(B_{n})$
24.                **Resampling**: $\left[{\left\{p_{s,n}^{r}\right\}}_{r=1}^{M_{r}}]=\mathrm{RESAMPLE}[{\left\{p_{s,n-1}^{r},w_{syn}^{s,r}\right\}}_{r=1}^{M_{r}}\right]$
**▷ Resampling Procedure**
25.                **for**
*r* = 1, …,*M_r_*
**do**▷ add Gaussian noise to avoid particle impoverishment26.                    Draw $\varepsilon_{r}∼N(0,\sigma_{n}^{2})$ and set $p_{s,n}^{r}=p_{s,n}^{r}+\varepsilon_{r}$27.                **end for**28.               **end for**
29.            **Select** randomly *D* seeds from current particle swarm **𝒫**_s,n_: 
**▷ Mean-Shift Clustering Procedure**
30.                $P_{s,n}^{sub}={\left\{p_{s,n}^{d}|p_{s,n}^{d}\in P_{s,n}\right\}}_{d=1}^{D}$

31.              **repeat** until convergence for each seed $p_{s,n}^{d}$:
32.                 **for**
*d* = 1, …, *D*
**do**▷ calculate shift vector iteratively and obtain cluster left according to expression (6)33.                    $\Delta p_{s,n-1}^{d}=\mathrm{mean}\_\mathrm{shift}(p_{s,n-1}^{d},w_{syn}^{s,r},b_{clust})$
34.                    $p_{s,n}^{d}=p_{s,n-1}^{d}+\Delta p_{s,n-1}^{d}$
35.                **end for**
36.            **end repeat**
37.            **Cluster** the particles and **obtain** max probability and state $\left\{Pr_{\max}^{s,n},p_{\max}^{s,n}\right\}$
38.            **if**
$Pr_{\max}^{s,n}$> *THR_pr_* and ${\left[p_{\max}^{s,n}\right]}_{\mathrm{str}}$>*THR*_str_
**then**▷ *THR*_pr_ and *THR*_str_ are the threshold about minimal probability and strength through which determine an effective cluster state39.                
$C_{s,n}=p_{\max}^{s,n}$
40.             else then41.                
$C_{s,n}=\phi$
42.             **end if**43.        **end for**44.        **for** m = 1, …, *N*
**do**
**▷ Confidence and State Estimation**
45.            **Compute** the dose rate given predicted cluster ${\left\{C_{s,n}\right\}}_{s=1}^{M_{s}}$ and position $I_{m}^{pos}$:46.                
$I_{m}^{\prime }=\mathrm{intensity}\_\mathrm{prediction}({\left\{C_{s,n}\right\}}_{s=1}^{M_{s}},I_{m}^{pos})$

47.        **end for**
48.        F = 0**▷** calculate the confidence of entire radiation field according to expression (13)49.        **for**
*m* = 1, …, *N*
**do**50.            
$F={\tilde{w}}_{obs}(I_{m}^{\prime },I_{m})/N+F$
51.        **end for**52.        **if**
F > F_best_
**then**
**▷ Configuration Maintenance**
53.            Update the best condition and corresponding state: ${\left\{C_{\mathrm{best}}^{s},P_{\mathrm{best}}^{s},F_{best}\right\}}_{s=1}^{M_{s}}$54.            count = 0
55.        **else then**
56.            count = count + 1
57.        **end if****▷** best configuration storing and controlling machine58.        **if** count > *THR*_count_
**then**59.            
${\left\{C_{s,n}\right\}}_{s=1}^{M_{s}}={\left\{C_{best}^{s}\right\}}_{s=1}^{M_{s}}$
${\left\{P_{s,n}\right\}}_{s=1}^{M_{s}}={\left\{P_{best}^{s}\right\}}_{s=1}^{M_{s}}$
**▷***THR*_count_ is the threshold whether to recover best configuration **▷**
*THR*_pr_end_ is the threshold whether to pass over expected probability60.            count = 061.        **end if**62.        **if** ℱ_best_ > *THR*_pr_end_
**then**63.            break the loop64.        **end if**
65.         Update active measurement set: I*_active_* = I*_active_* – *I_rand(n)_*
67.    **end for**
68.
**end for**

69.**if** ℱ_best_ > *THR*_pr_end_
**then**▷ determine whether it’s an effective prediction by threshold *THR*_pr_end_70.    
A=ϕ
71.    **for**
*s* = 1, …, *M_s_*
**do**72.        **if**
$C_{s,n}\neq \phi$
**then**
73.             Append *C_s_*_,*n*_ to 𝒜▷ append non-empty cluster states to the predicted set 𝒜74.        **end if**75.    **end for**
76.
**else then**
▷ append empty set to 𝒜, meaning prediction failed77.      
A=ϕ
78.
**end if**

79.**Output** predicted radiation sources state 𝒜.
**▷ Output Predicted Sources**


## 5. Simulation and Experimental Research

In this section, simulations and real-world experiments are completed to evaluate the effectiveness and performance of the PSPF algorithm. To measure the prediction accuracy of the proposed method, the deviations about source localization and radiation intensity are simulated and carefully documented in different experimental factors, e.g., background radiation level, number of radiation sources and processing runtime, etc. It should be noted that the measuring positions in simulations are extracted from the real-world detection set, for the sake of comparison and validating the algorithm in practice. The environment and scenario settings are listed in Table 1.

### 5.1. Simulation Scene 1: Localization with Different Background Radiation

**Figure 6:**
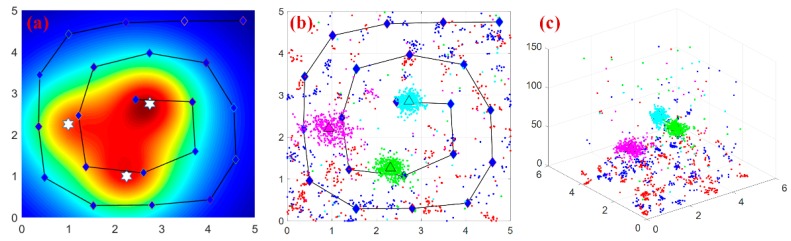
The simulation scenario for 3 radiation sources localization. (**a**) The cloud chart of theoretical mixed radiation field. (**b**,**c**) The simulation interface and estimated results of the five-layer PSPF algorithm (2D & 3D).

**Figure 7:**
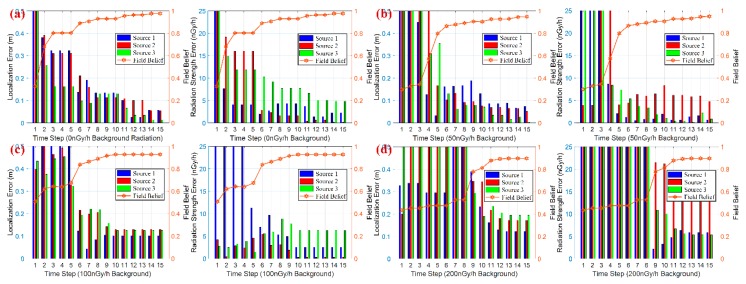
Localization and strength error with different background radiation. (**a**–**d**) respectively illustrates the estimation results with background radiation level of 0, 50, 100 and 200 nGy/h.

The simulation interface and estimation scenario for three radiation sources is illustrated in Figure 6. As the background radiation is 100~200 nGy/h in general, four simulation groups with three radiation sources and different background radiation level are completed to evaluate the algorithm robustness, as shown in Table 1. Considering the stochastic feature, Gaussian noise with predefined background as mean whilst half background as standard deviation is attached to the sensor measurements. Each simulation case has been repeated for 10 times and average performance of the algorithm is illustrated in Figure 7.

Figure 7 presents the results of three sources localization with different background levels, and the overall field belief for all sensor measurements reaches 97.62%, 94.88%, 92.73% and 89.65% respectively.

The results analysis is listed in the following:

(i) Numeric simulations show that PSPF algorithm works well in the above four conditions, i.e., 0, 50, 100 and 200 nGy/h background radiation for each case. However, more iterations are required to produce accurate prediction as the background increases. Especially with the 200 nGy/h background level (e.g., the noise almost accounts for 20% of the signal), 8 iterations are taken to produce a relatively stable and accurate estimates.
(ii) In the term of false positives and negatives, all the estimates correspond to the correct sources, that is, no more or less cluster centers are produced in each simulation. As 5 candidate particle swarms are employed for estimation, it can be observed that two particle swarms remain non-clustered status along the whole prediction process. The phenomenon and simulation results validate the non-parametric property about source number determination in the proposed algorithm.
(iii) From above simulation results, we can see the final confidence score increases to a high level even in large background noise, validating the robustness and practicability of the algorithm. In addition, a great improvement on field belief can be observed after several iterations, while the remaining procedure is just local correction on candidate parameters. This fact indicates the necessity of the iteration over sensor measurements.

### 5.2. Simulation Scene 2: Large-Scale Multiple Sources Estimation

The scalability of source number is an important indicator for any localization algorithm without prior sources knowledge. In our case, the number of required particles maintains a linear relationship with the particle swarm number, rather than growing exponentially as the source number increases. The linear computational complexity is due to the sequential particle swarm estimation and non-parametric property about source number.

**Figure 8:**
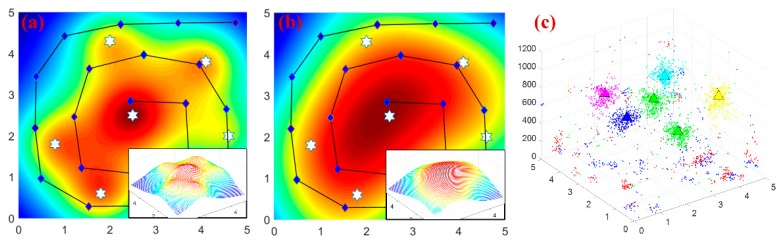
The simulation scenario for 6 radiation sources localization. (**a**) Theoretical radiation strength distribution. (**b**) Radiation strength distribution with simply GPs regression. (**c**) The estimation results of the multiple-layer PSPF algorithm.

This scenario illustrates the scalable performance for 6 actual sources and 8 particle swarms with 100 nGy/h noise in the surveillance area, which would cause analytical intractability in other existing algorithms. The simulation layout and estimation scenario are shown in Figure 8. In particular, all the measurements (only 21 points) are regressed simply with Gaussian Processes Regression to approach the actual radiation field (as Figure 8b). Evidently, the simple regression method fails to locate the multiple radiation sources with sparse measurements, and that’s the purpose of developing the PSPF algorithm.

**Figure 9:**
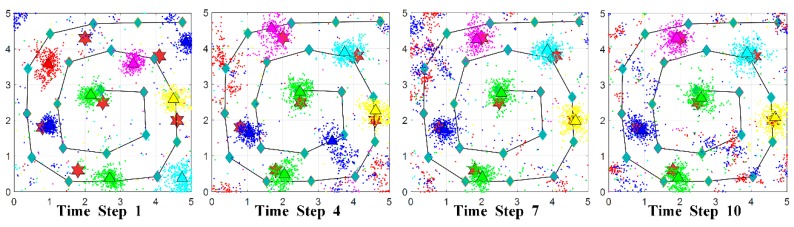
Progression of PSPF estimation for 6 radiation sources localization over time.

**Figure 10:**
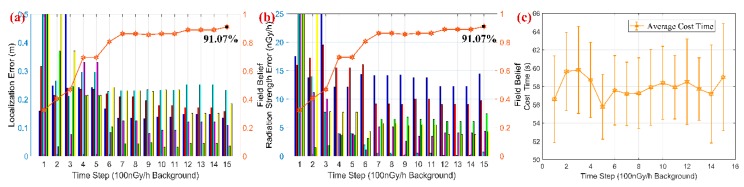
The results of large-scale sources scenario. (**a**,**b**) illustrates the estimation error and overall.

Figure 9 shows the progression of the PSPF algorithm for large-scale sources localization (6 radiation sources and 8 particle swarms). The estimation error and confidence score are presented in Figure 10. It can be seen that the state particles gradually cluster and move toward the actual radiation parameters, and two state particle swarms always remain non-clustered status all along the process. Additionally, quantitative analysis in Figure 10 indicates that confidence score goes steadily up whilst the convergence for source estimates works well over time, and the final confidence belief reaches 91.07%. Although the confidence score declines a little in comparison with three sources case (as shown in Section 5.3), above facts validate the excellent performance in large-scale sources localization, i.e., the number scalability of the PSPF method. Figure 10c indicates that the proposed algorithm progresses smoothly and the running time for each step is around 58 s, meaning 0.35 s per particle swarm estimation.

### 5.3. Simulation Scene 3: Processing Runtime Test

**Figure 11:**
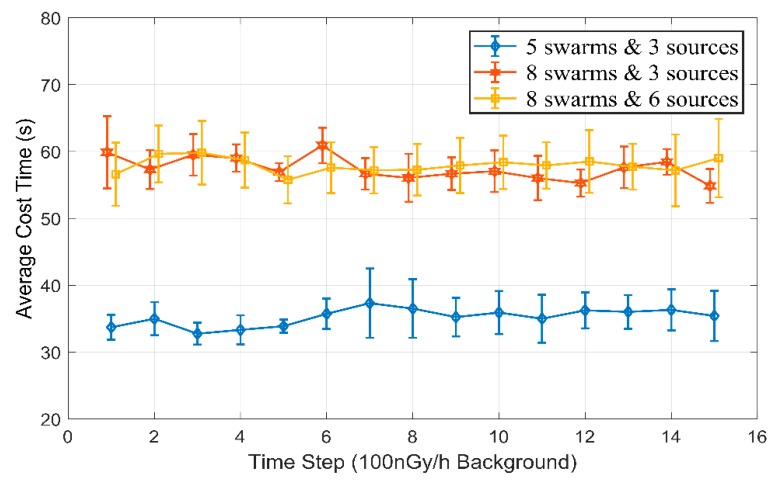
The processing runtime with different swarm number and source number cases.

As processing runtime is important for dimensional scalability and calculative tractability of the proposed algorithm, three experimental groups with different swarm number and source number are completed in this scenario, to explore the related factors processing runtime. The settings of swarm number and source number are (8,6), (8,3) and (5,3) respectively. The simulation is performed on a personal computer with 2.60 GHz Intel Core i5 and 4 GB RAM, the average runtime and two times standard deviation for three cases are presented in Figure 11.

As illustrated above, the statistical analysis of this scenario mainly explores the relationship among running time, particle swarms number and actual radiation source number. By comparison of different cases, we can obtain two conclusions:

(i) Processing runtime are roughly linear to the pre-defined particle swarms number, while the number variation of actual radiation sources nearly does not change the estimation duration. The linear complexity with swarm number benefits from application of the multi-layer swarm structure, which transforms dimensional scalability into swarm number scalability.
(ii) The average processing speed for particle swarm prediction is respectively 0.345 s, 0.342 s and 0.336 s in three cases, that is, the prediction period for one swarm estimation is almost the same. The phenomenon demonstrates once again that PSPF algorithm can handle the multi-sources localization problem in a linear complexity with the pre-estimated particle swarm number. Additionally, this fact also indicates that further timeliness research may focus on the operations in the swarm estimation procedure.

### 5.4. Field Experiment for Two Radiation Sources Localization

**Figure 12:**
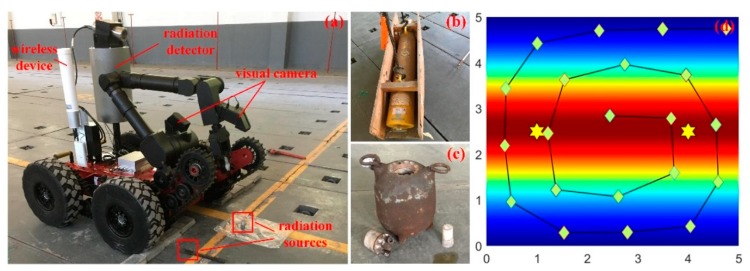
The localization experiment for two radiation sources. (**a**) is the global scene of the experiment. (**b**,**c**) shows the two radiation sources and corresponding containers respectively. (**d**) illustrates the source locations, sampling points and measurements cloud chart with GPs regression.

The PSPF algorithm has been experimentally verified utilizing Geiger-Muller counter mounted on a mobile manipulator, and the self-developed mobile platform is also equipped with 7-DOF manipulator, wireless device, wheel odometer and visual cameras, as shown in Figure 12a. The GM tube (70031, VACUTEC) can capture the ionizing energy ranging from 35 KeV~1.3 MeV, and the conversion constant Ei provided by manufacturer is 1/16, i.e., 16*CPS* equals to 1 μGy/h. Considering the health hazards of radioactive rays, two point sources with low dose rate (100 μCi, Co-60) were employed for the localization experiment, which was carried out in a radiation sources repository (one radiation source is welded in the steel stick). These two radiation sources were placed in 3 m apart from each other, i.e., located at (1.0, 2.5) and (4.0, 2.5) in the surveillance area. Furthermore, the radiation distribution was predicted simply with GPs method in advance, which was unable to identify the source locations with sparse measurements, as illustrated in Figure 12d. As referenced in [32], a spiral detection trajectory was adopted for better detection efficiency and area coverage, which is also employed in previous simulations for the sake of comparison.

**Figure 13:**
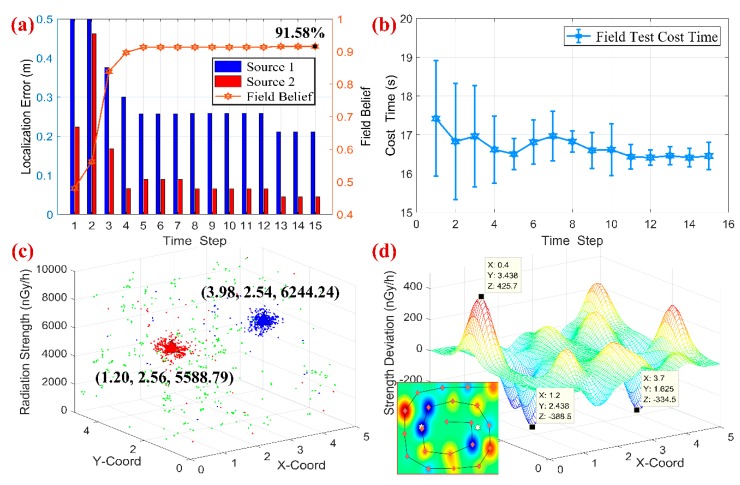
Quantitative analysis of field experiment. (**a**) the localization error and field belief w.r.t. time steps. (**b**) the cumulative cost time for each time step. (**c**) the final estimated clusters. (**d**) the regression surface of the measurements deviation.

Together with these sparse measurements and three particle swarms, the PSPF algorithm was performed for the field localization scenario, and the background dose rate could be obtained through the handheld-type radiation detector, i.e., the dose rate ranging from 260 nGy/h~290nGy/h. The proposed state estimation method was eventually carried out with 275 nGy/h background radiation, and the quantitative analysis is presented in Figure 13, including localization error, overall field belief and the regression surface about dose measurements deviation.

As shown in Figure 13a,b, the estimation procedure enters the steady phase in 5 time steps, when the clustered estimates are (1.20, 2.56, 5588.79) and (3.98, 2.54, 6244.24), and the overall confidence score reaches to 91.31%. We can obtain several conclusions from the field experiment: (i) Even with sparse measurements (21 points), accurate estimates could still be obtained, and the non-parametric property about number of radiation sources also works well. The prediction results validate the effectiveness and practicability of the PSPF algorithm. (ii) Figure 13d shows the regressive surface of detection deviation between actual measurements and estimation results. It could be seen that the deviations located close to sources are always negative while the ones far away are positive, which is due to the occlusion of the mobile platform. Furthermore, the maximum residual (427.5 nGy/h) nearly takes up 10% of the radiation strength. The violent fluctuation of the surface verifies the robustness of PSPF method in another aspect. (iii) The total runtime of PSPF algorithm is about 260 s (15 time steps), meaning 0.275 s for each sensor reading per particle swarm. This processing speed is sufficient for the multiple sources localization task.

## 6. Conclusions

In this paper, a novel particle filter based method is conceived and developed to handle the multimodal estimation problem in mixed radiation field with sparse measurements. The PSPF algorithm constructed combines sequential multi-layer estimation and peak suppression technique to achieve the multimodal maintenance of the particle swarms. Benefiting from the multiple swarms setting and specific centroid filtering criteria, the non-parametric property about source number has be obtained and verified through simulations and experiments. Additionally, a distance correction model and a configuration restoring mechanism have been conceived and implemented, improving the stability and convergent performance of the algorithm. Simulations and field experiment have been conducted and verified in several aspects, i.e., background radiation level, radiation sources number, processing speed and non-parametric property, all the experimental results have validated the robustness and practicability of the PSPF method. It should be noted that our algorithm tackles with the multiple sources localization problem in cross-mixed radiation field, with excellent features of non-parametric and large-scale estimation about source number. Our future work is to establish a dynamic regulation mechanism to further improve the prediction accuracy and convergent speed.

## Figures and Tables

**Table 1:** The simulation settings with different scenarios.

Simulation Background		Simulation Setting
• size of surveillance area		5 m × 5 m
• origin of detection set		real-world measurements (spiral shape)
• range of sources strength		0~1500 nGy/h
• number of particles in each swarm		300
Scene 1	• number of particle swarms	5
	• number of radiation sources	3
	• parameters about sources	(1,2.25,790), (2.25,1,880), (2.75, 2.75,970)
	• simulation change factor	background radiation level (with 0, 50, 150, 250 nGy/h)
Scene 2	• number of particle swarms	8
	• number of radiation sources	6
	• parameters about sources	(0.8,1.8,680), (2.5,2.5,870), (4.1,3.8,720), (2.0,4.3,670), (4.6,2.0,565), (1.8,0.6,820)
	• simulation change factor	large number of radiation sources
Scene 3	• number of particle swarms	5/8
	• number of radiation sources	3/6
	• parameters about sources	similar to scene 1/scene 2
	• simulation change factor	processing runtime
